# Role of Rutin in 5-Fluorouracil-Induced Intestinal Mucositis: Prevention of Histological Damage and Reduction of Inflammation and Oxidative Stress

**DOI:** 10.3390/molecules25122786

**Published:** 2020-06-17

**Authors:** Lázaro de Sousa Fideles, João Antônio Leal de Miranda, Conceição da Silva Martins, Maria Lucianny Lima Barbosa, Helder Bindá Pimenta, Paulo Vitor de Souza Pimentel, Claudio Silva Teixeira, Marina Alves Sampaio Scafuri, Samuel de Osterno Façanha, João Erivan Façanha Barreto, Poliana Moreira de Medeiros Carvalho, Ariel Gustavo Scafuri, Joabe Lima Araújo, Jefferson Almeida Rocha, Icaro Gusmão Pinto Vieira, Nágila Maria Pontes Silva Ricardo, Matheus da Silva Campelo, Maria Elenir Nobre Pinho Ribeiro, Gerly Anne de Castro Brito, Gilberto Santos Cerqueira

**Affiliations:** 1 Department of Morphology, Faculty of Medicine, Federal University of Ceará, s/n Delmiro of Farias Street, Porangabuçu Campus, Fortaleza 60416-030, Brazil; lazarofideles@gmail.com (L.d.S.F.); josycristophe@hotmail.com (C.d.S.M.); marialucianny@gmail.com (M.L.L.B.); hbinda@uea.edu.br (H.B.P.); paulo_vitordesouza@hotmail.com (P.V.d.S.P.); claudioanatomia@yahoo.com.br (C.S.T.); erivanfacanha@yahoo.com.br (J.E.F.B.); urologia@gmail.com (A.G.S.); gerlybrito@hotmail.com (G.A.d.C.B.); giufarmacia@hotmail.com (G.S.C.); 2 Scafuri Institute of Human Sexuality, 1513 Republic of Lebanon Street, Varjota, Fortaleza 60175-222, Brazil; drmarinalvess@gmail.com; 3 Christus University Center (Unichristus), 133 Adolfo Gurgel Street, Fortaleza 63010-475, Brazil; samuelosterno@hotmail.com; 4 University Center of Juazeiro do Norte (UNIJUAZEIRO), 1224 San Francisco Street, Juazeiro do Norte-CE 63010-475, Brazil; polyfarma2004@yahoo.com.br; 5 Department of Genetics and Morphology, s/n Darcy Ribeiro University Campus, University of Brasília, Brasília-DF 70910-900, Brazil; joabearaujobiotec@gmail.com; 6 Medicinal Chemistry and Biotechnology Research Group (QUIMEBIO), Federal University of Maranhão (UFMA), São Bernardo/MA 65550-000, Brazil; ja.rocha@ufma.br; 7 Technological Development Park, Federal University of Ceará, Humberto Monte Avenue, 2977, Pici Campus, Fortaleza 60440-900, Brazil; icarogpv@uol.com.br; 8 Department of Organic and Inorganic Chemistry, Federal University of Ceará, Pici Campus, Fortaleza 60440-900, Brazil; naricard@ufc.br (N.M.P.S.R.); matheus.campelo@hotmail.com (M.d.S.C.); elenir.ribeiro@ufc.br (M.E.N.P.R.)

**Keywords:** flavonoid, inflammation, antimetabolites, intestine

## Abstract

Intestinal mucositis, characterized by inflammatory and/or ulcerative processes in the gastrointestinal tract, occurs due to cellular and tissue damage following treatment with 5-fluorouracil (5-FU). Rutin (RUT), a natural flavonoid extracted from *Dimorphandra gardneriana*, exhibits antioxidant, anti-inflammatory, cytoprotective, and gastroprotective properties. However, the effect of RUT on inflammatory processes in the intestine, especially on mucositis promoted by antineoplastic agents, has not yet been reported. In this study, we investigated the role of RUT on 5-FU-induced experimental intestinal mucositis. Swiss mice were randomly divided into seven groups: Saline, 5-FU, RUT-50, RUT-100, RUT-200, Celecoxib (CLX), and CLX + RUT-200 groups. The mice were weighed daily. After treatment, the animals were euthanized and segments of the small intestine were collected to evaluate histopathological alterations (morphometric analysis); malondialdehyde (MDA), myeloperoxidase (MPO), and glutathione (GSH) concentrations; mast and goblet cell counts; and cyclooxygenase-2 (COX-2) activity, as well as to perform immunohistochemical analyses. RUT treatment (200 mg/kg) prevented 5-FU-induced histopathological changes and reduced oxidative stress by decreasing MDA concentrations and increasing GSH concentrations. RUT attenuated the inflammatory response by decreasing MPO activity, intestinal mastocytosis, and COX-2 expression. These results suggest that the COX-2 pathway is one of the underlying protective mechanisms of RUT against 5-FU-induced intestinal mucositis.

## 1. Introduction

The use of chemotherapeutic agents is a major strategy for destroying cancer cells and maintaining normal cell development in cancer patients [1]. 5-Fluorouracil (5-FU), an antimetabolic chemotherapeutic agent used mostly to treat various types of cancer and tumors, is a fluorinated pyrimidine whose anticancer activity is based on its capacity to interfere with nucleotide metabolism, as well as RNA and DNA synthesis [2,3,4,5]. However, several adverse effects are associated with 5-FU therapy, including nausea, vomiting, diarrhea, myelosuppression, and intestinal mucositis [6,7,8,9,10,11].

Intestinal mucositis is characterized by inflammatory and/or ulcerative processes in the gastrointestinal tract, resulting in cellular and tissue damage caused by 5-FU treatment [12]. Notably, the incidence of mucositis in cancer patients ranges between 40 and 100% [13,14,15].

Owing to the lack of efficacious therapeutic tools for the treatment of intestinal mucositis, new alternative therapeutics that can reduce the side effects of 5-FU, without impairing cancer treatment, are being studied.

Numerous studies have explored secondary metabolites from medicinal plants such as flavonoids with the aim of finding metabolites that exhibit pharmacological activity against several human health disorders [16]. Several flavonoids with antispasmodic, antisecretory, antidiarrheal, and antiulcer properties within the gastrointestinal tract have been reported [17,18]. For these flavonoids, their biological activities are associated with their capacity to modulate key enzymes involved in the stimulation of inflammatory, oxidative, and catabolic processes, including xanthine oxidase (XO), cyclooxygenase (COX), lipoxygenase, and phosphoinositide 3-kinases [19].

Rutin (quercetin-3-rutinoside, RUT), a natural flavonoid extracted from *Dimorphandra gardneriana*, Fabaceae, popularly known as anta fava [20]. *D. gardneriana* is an extremely abundant plant in the Brazilian biome, naturally occurring in the northeastern region of the country [21] and has been widely used by the pharmaceutical industry in Brazil to extract RUT [22]. For RUT, a phytochemical with superior therapeutic potential, it has been reported to exhibit antioxidative [23], anti-inflammatory [24], anti-apoptotic and cytoprotective [25,26,27] activities, as well as gastroprotective efficacy [28,29]. However, the effect of RUT on inflammatory processes in the intestine, especially on mucositis promoted by antineoplastic agents, has not yet been reported. In this study, we investigated the role of RUT on experimental 5-FU-induced intestinal mucositis and explored the possible underlying mechanisms of its action.

## 2. Results

### 2.1. Extraction and Characterization of the RUT Flavonoid

The chromatographic fractionation of *D. gardneriana* extracts facilitated the isolation of a yellow crystalline solid with a melting point of 194 °C (with decomposition), homogeneous in thin layer chromatography (TLC) and soluble in methanol and dimethyl sulfoxide (DMSO). The method resulted in the purification of 12.42 g RUT.

**Figure 1:**
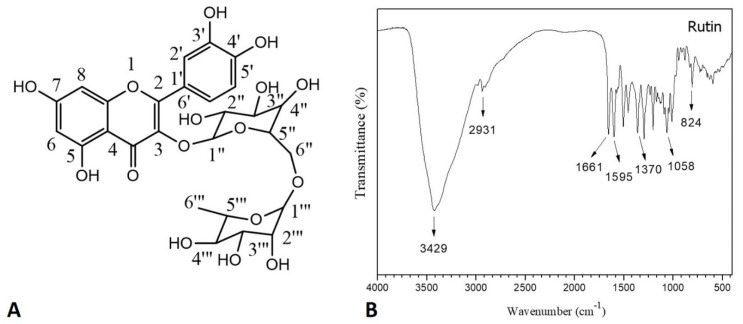
Characterization of Rutin (RUT). (**A**) Chemical structure of RUT; (**B**) Absorption spectroscopy in the infrared region of RUT.

Based on the infrared spectrum analysis of RUT (Figure 1B), it was possible to verify the major functional groups present in its chemical structure (Figure 1A). The broad and strong band at 3429 cm^−1^ indicated the stretching of the O-H bond of the hydroxyl groups, indicating alcohol and phenol chemical structures. The band observed at 2931 cm^−1^ was attributed to the asymmetric stretch of the C-H bond. Additionally, the doublet present between 1661 and 1595 cm^−1^ indicated the stretching of the C=O bond of the carbonyl function and the C=C bond of the aromatic rings, respectively.

The band present at 1370 cm^−1^ indicated the vibrations of the C-O bond, while the band present at 1058 cm^−1^ indicated the stretching of the C-O-C bond, as well as the presence of conjugated carbohydrates in the chemical structure of the molecule. Moreover, absorption at 824 cm^−1^ indicated a substituted aromatic ring. The assignments are consistent with those reported by Vu et al. [30] and Deepika et al. [31].

**Figure 2:**
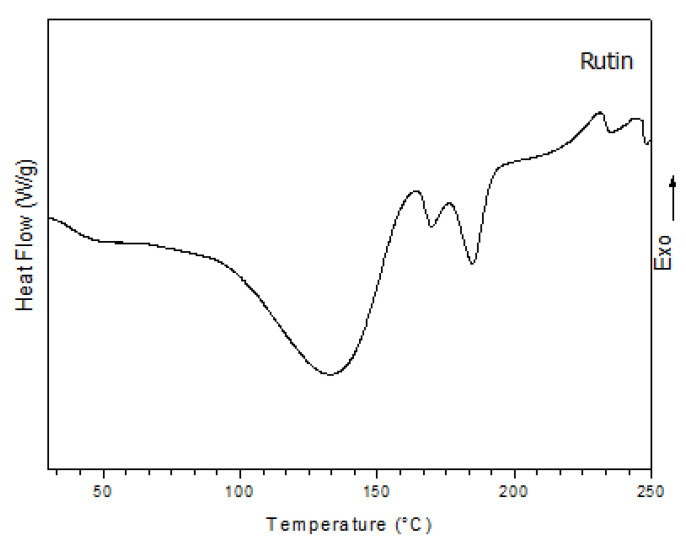
Differential Scanning Calorimetry (DSC) curve of RUT under N_2_ atmosphere.

The Differential Scanning Calorimetry (DSC) curve of RUT isolated from *D. gardneriana* (Figure 2) showed the presence of three endothermic peaks ranging from 134 to 185 °C, and two exothermic peaks at 230 and 244 °C. The peak at 134 °C refers to the loss of residual water present in the physical structure of the drug. The melting temperature refers to the peaks between 169 and 185 °C. The exothermic peaks at 230 and 244 °C are attributed to the boiling temperatures.

The Carbon-13 Nuclear Magnetic Resonance (^13^C-NMR) spectrum of RUT isolated from *D. gardneriana* demonstrated 27 spectral lines facilitating the identification of 15 absorptions associated with unsaturated carbons in the δ 177.8–94.0 range and 12 absorptions associated with saturated carbons in the δ 122.0–18.2 range when compared with the ^13^C-distortionless enhancement of polarization transfer DEPT-NMR spectra (135°). Furthermore, the proton (^1^H) NMR spectrum revealed hydroxyl groups from a singlet at δ 12.6, indicating the presence of four absorptions associated with unsaturated carbon hydrogens, demonstrating chemical displacements in the δ 7.5–6.2 range. The assignments are listed in Table 1 and are in line with the proposals of Xiao et al. [32] and Xu et al. [33].

**Figure 3:**
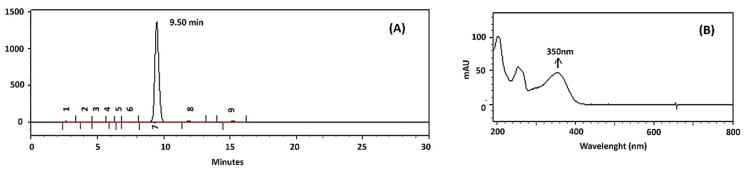
HPLC chromatogram (**A**) and UV-Vis spectrum (**B**) of the isolated RUT from *D. gardneriana.*

Figure 3 shows the High Performance Liquid Chromatography (HPLC) chromatogram (A) and the absorption spectrum in the UV-Vis region of the isolated RUT from *D. gardneriana* (B). The isolated RUT had a retention time of 9.50 min and a purity of 97% HPLC grade. In addition, the scanning spectrum in the UV-Vis region indicated a maximum absorbance peak at 350 nm.

### 2.2. Weight Analysis

**Figure 4:**
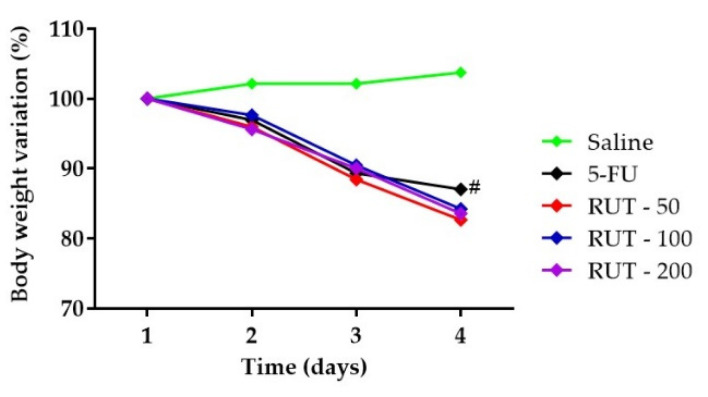
Body weight variation in mice subjected to induced intestinal mucositis (5-FU, 450 mg/kg, intraperitoneally, single dose) and treated with RUT (50, 100, and 200 mg/kg for 3 days). The results are expressed as the mean ± standard error of the mean (SEM) of the weight evaluation percentage of the initial weight, for a minimum of 6 animals per group. Statistical analysis was performed using two-way analysis of variance (ANOVA) followed by Tukey’s test. # *p* < 0.05 vs. saline.

Weight loss is one of the most common side effects of 5-FU chemotherapy. The experimental mouse model of 5-FU-induced intestinal mucositis demonstrated significant weight loss. As shown in Figure 4, from the second day, all mice with 5-FU-induced intestinal mucositis presented progressive weight loss, which was significant when compared to the saline group (*p* < 0.05). However, pretreatment with RUT at any dose failed to prevent weight loss in the 5-FU-induced mucositis animals (*p* < 0.05).

### 2.3. Histopathological and Morphometric Analysis

As shown in Table 2, the administration of 5-FU induced changes in the intestinal mucosa of mice, as evidenced by reduced villus height, crypt necrosis and hypoplasia, intense inflammatory cell infiltration, vacuolization, and edema of intestinal mucosal and muscular layer cells. In the 5-FU-administered group, this resulted in a significant increase in the microscopic score when compared to the saline group (*p* < 0.05) in the three intestinal segments. Notably, RUT treatment (200 mg/kg) significantly decreased (*p* < 0.05) the histopathological scores when compared to the 5-FU lesion group, demonstrating the reversal of decreased villus height and vacuolization, as well as the attenuation of crypt necrosis and inflammatory cell infiltration into the intestinal mucosa in the duodenal and jejunal segments. In the three intestinal segments, RUT (50 and 100 mg/kg) demonstrated no significant decrease (*p* > 0.05) in the histopathological scores induced by 5-FU.

**Figure 5:**
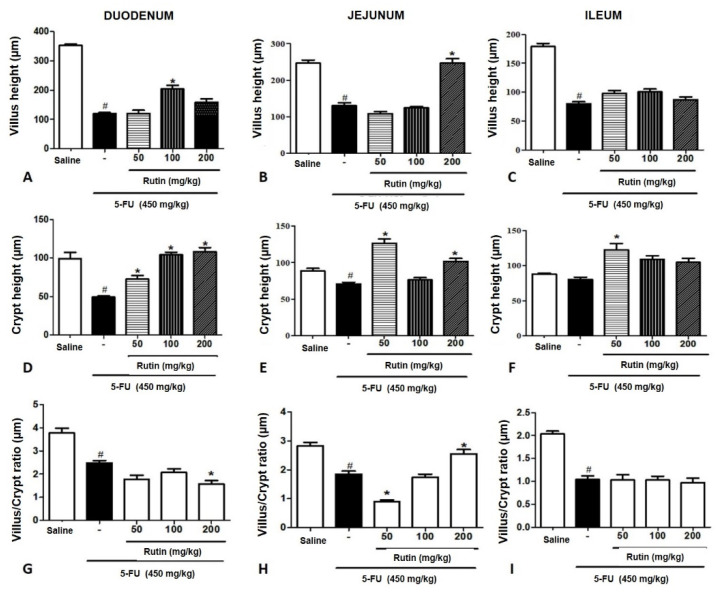
Morphometric analyses of the mice intestinal segments following 5-FU-induced intestinal mucositis. Experimental groups: Saline, 5-FU, RUT-50, RUT-100, and RUT 200 mg/kg. (**A**–**C**): Villi height in the duodenal, jejunal, and ileal segments, respectively. (**D**–**F**): Crypt depth of the duodenal, jejunal, and ileal segments, respectively. (**G**–**I**): Villus/crypt ratio of the duodenal, jejunal, and ileal segments, respectively. Values are expressed as mean ± SEM. Statistical analysis was performed using the one-way analysis of variance, followed by Tukey’s test. # *p* < 0.05 vs. saline group and * *p* < 0.05 vs. group 5-FU. Villus height (µm), Crypt height (µm), Villus/Crypt ratio (µm). 5-FU, 5-fluorouracil; RUT, rutin.

Additionally, the villi heights were measured in the three intestinal segments (Figure 5A–C); 5-FU significantly decreased the villi height in the duodenum, jejunum, and ileum (*p* < 0.05, when compared to the saline group in corresponding segments. In the duodenum and the jejunum, pretreatment with RUT (100 and 200 mg/kg) revealed a significant reversal in villi shortening induced by 5-FU (*p* < 0.05). However, in the ileum, all RUT doses failed to reverse the villi shortening caused by 5-FU treatment.

We demonstrated that 5-FU significantly decreased crypt depths in the duodenal segment when compared with the depths in the saline group; RUT 50, 100, and 200 mg/kg prevented the decrease in crypt depths when compared with the 5-FU treatments (*p* < 0.05). Additionally, in the jejunum, a significant decrease in crypt depths was induced by 5-FU (*p* < 0.05) treatment when compared to the crypts in the saline group; RUT 50 and 200 mg/kg reversed the decreases in crypt depth induced by 5-FU. In the ileum, no significant differences (*p* < 0.05) were observed in crypt depths between the 5-FU and saline groups.

According to the results of the villus/crypt ratio analyses among the three intestinal segments (Figure 5G–I), the villus/crypt ratio in the duodenum decreased significantly following treatment with 5-FU when compared with the saline group (*p* < 0.05). No RUT treatment increased the villus/crypt ratio significantly when compared with the ratio in the 5-FU group. In the jejunum, 5-FU decreased villus/crypt ratio significantly when compared with the saline group (*p* < 0.05), and treatment with RUT 200 mg/kg could reverse the decrease in villus/crypt ratio promoted by the 5-FU (*p* < 0.05). In the ileum, 5-FU decreased the villus/crypt ratio significantly when compared with the saline group (*p* < 0.05); however, none of the tested RUT doses promoted a significant increase (*p* < 0.05) in villus/crypt ratio when compared to the 5-FU group.

**Figure 6:**
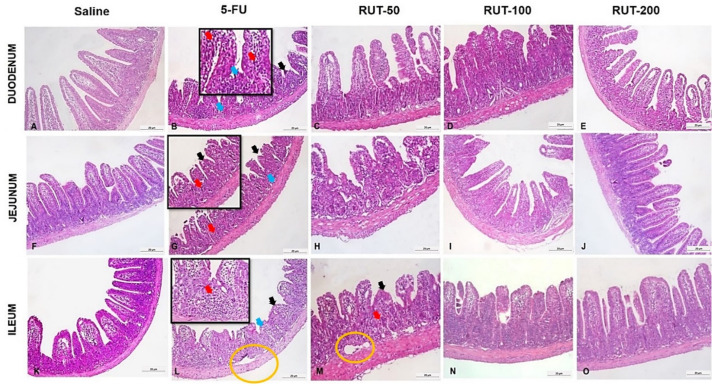
Histopathological analyses of intestinal segments of mice with 5-FU-induced intestinal mucositis. Horizontally, the duodenum is illustrated in (**A**–**E**), corresponding to the saline, 5-FU, RUT 50, 100, and 200 mg/kg groups, respectively. The jejunum is illustrated in (**F**–**J**), representing the saline, 5-FU, RUT 50, 100, and 200 mg/kg, groups, respectively. The ileum is illustrated in (**K**–**O**), representing the saline, 5-FU, RUT 50, 100, and 200 mg/kg, respectively. Black arrows: Shortened villi; red arrows: inflammatory infiltrates, blue arrows: loss of crypt architecture, and yellow circle: edema. In all segments, 5-FU induces shortened villi, loss of crypt architecture, and intense inflammatory infiltration. Histological changes are notably reduced following pretreatment with RUT 200 mg/kg, especially in the duodenal and jejunal segments. 5-FU, 5-fluorouracil; RUT, rutin.

Figure 6 illustrates that 5-FU reduced villi heights, and induced necrosis, loss of crypt architecture and an increase in the inflammatory infiltrate when compared with observations in the saline group. The results suggest that RUT prevented histological alterations induced by 5-FU treatment, and its protective effects on the jejunal segment and the effects of 200 mg/kg RUT were particularly evident.

### 2.4. Myeloperoxidase Assay (MPO)

**Figure 7:**
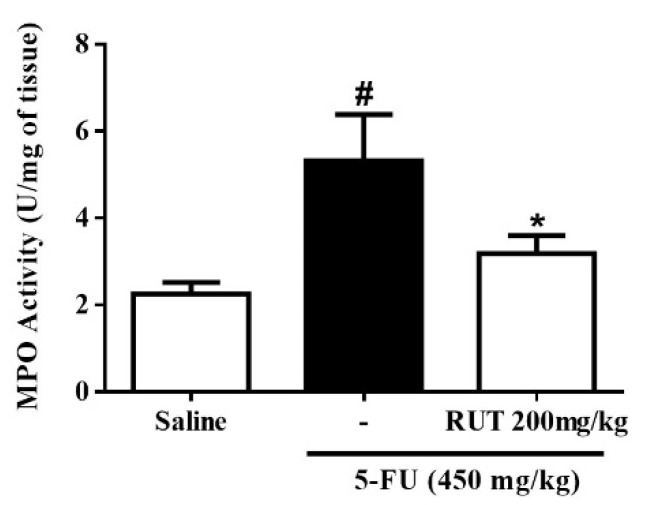
Myeloperoxidase (MPO) assay. Values are presented as mean ± SEM of MPO activity expressed in Unit (U) of MPO/mg in tissue. Statistical analysis was performed using the one-way analysis of variance followed by the Tukey’s test. # *p* < 0.05 vs. saline group and * *p* < 0.05 vs. group 5-FU.

To investigate the effects of RUT pretreatment on neutrophil recruitment in 5-FU-induced intestinal mucositis, we evaluated the levels of myeloperoxidase (MPO), a neutrophil marker, in the jejunum. As shown in Figure 7, 5-FU significantly increased MPO levels per mg of tissue in the jejunal segment when compared to the saline group (*p* < 0.05). Conversely, RUT 200 mg/kg significantly decreased MPO levels when compared to the 5-FU group (*p* < 0.05) and, therefore, decreased neutrophil infiltration (Figure 7).

### 2.5. Malondialdehyde (MDA) and Glutathione (GSH) Levels

**Figure 8:**
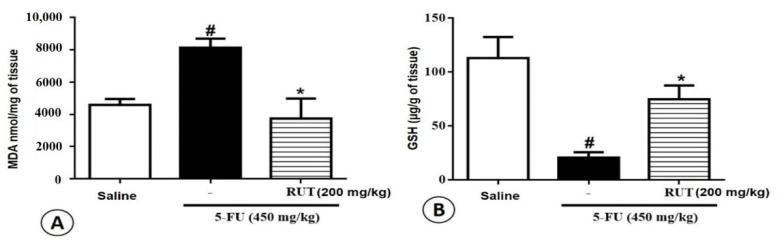
Analysis of oxidative stress. (**A**) Malondialdehyde (MDA), (**B**) Glutathione (GSH). Experimental groups: Saline, 5-FU, and RUT 200 mg/kg. Values are presented as mean ± SEM. For the statistical analyses, one-way analysis of variance was used, followed by Tukey’s test. # *p* < 0.05 vs. saline group and * *p* < 0.05 vs. group 5-FU. 5-FU, 5-fluorouracil; RUT, rutin.

To investigate the effects of RUT pretreatment on 5-FU-induced oxidative stress in the jejunum, MDA and GSH levels (final products of oxidative stress) were evaluated. In the jejunum, 5-FU treatment increased MDA levels when compared to those observed following saline treatment (*p* < 0.05, Figure 8A). Administration of 200 mg/kg RUT decreased MDA concentrations when compared to the concentrations observed under the 5-FU treatment (*p* < 0.05). Animals treated with 5-FU exhibited significantly decreased GSH concentrations when compared with animals in the saline group (*p* < 0.05). However, GSH levels increased significantly in the RUT 200 mg/kg group when compared with levels in the 5-FU group (*p* < 0.05, Figure 8B).

### 2.6. Cell Count in the Intestinal Mucosa: Mast and Goblet Cells

**Figure 9:**
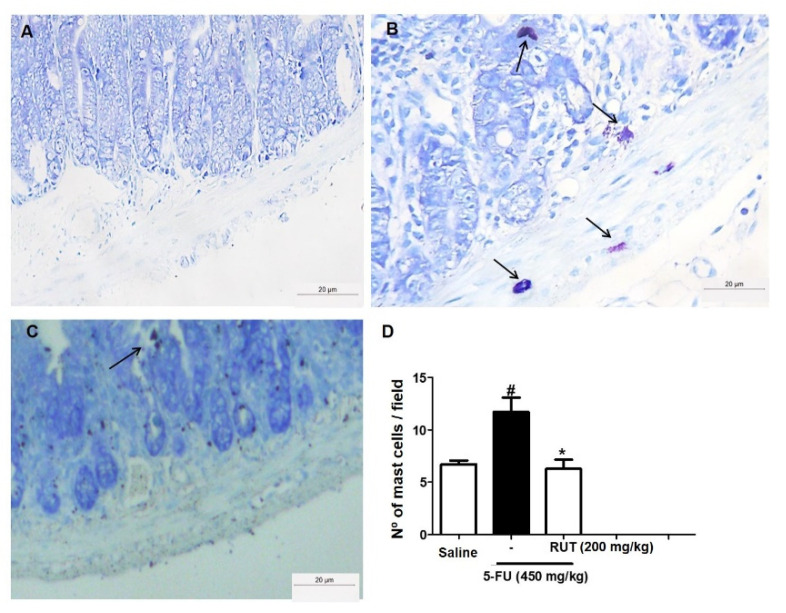
Mast cell counts in the jejunal segment based on Toluidine Blue staining. Saline (**A**); 5-FU (**B**); RUT-200 (**C**); Statistical representation of experimental groups (**D**). Black arrows indicate mast cells. All the panels were obtained at 400× magnification. Values are presented as mean ± SEM of the number of mast cells per field. For the statistical analysis, one-way ANOVA was used, followed by Tukey’s test. # *p* < 0.05 in 5-FU vs. Saline group; * *p* < 0.05 in RUT-200 vs. 5-FU group. 5-FU, 5-fluorouracil; RUT, rutin.

To evaluate the effect of RUT pretreatment on 5-FU-induced mastocytosis, the number of mast cells in the jejunum was determined (Figure 9). 5-FU treatment significantly increased the number of mast cells per field when compared with saline treatment (*p* < 0.05). Additionally, the administration of 200 mg/kg RUT (Figure 9C) reduced the number of mast cells in turn, preventing 5-FU-induced mastocytosis and degranulation in mouse intestines (Figure 9B,D).

**Figure 10:**
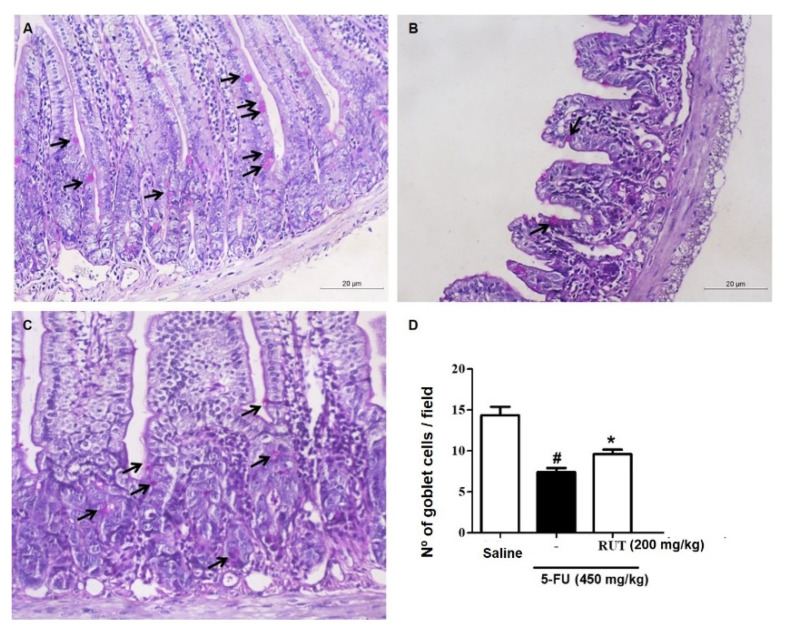
Goblet cell counts in the jejunal segment based on Schiff periodic acid (PAS) staining. Saline (**A**); 5-FU (**B**); RUT-200 (**C**); Statistical representation of experimental groups (**D**). Black arrows indicate goblet cells. Values are presented as mean ± SEM for the number of goblet cells per field. For the statistical analysis, one-way ANOVA was used, followed by Tukey’s test. # *p* < 0.05 in 5-FU vs. Saline group; * *p* < 0.05 in RUT-200 vs. 5-FU group. 5-FU, 5-fluorouracil; RUT, rutin.

Analysis of goblet cells in the jejunal segment (Figure 10) revealed that 5-FU treatment significantly decreased the number of goblet cells in the intestinal mucosa (*p* < 0.05) when compared to saline treatment. However, treatment with 200 mg/kg RUT retained the goblet cell number (Figure 10C), when compared with that of 5-FU treatment (*p* < 0.05, Figure 10B,D).

### 2.7. Effect of RUT on Cyclooxygenase-2 Pathway Based on Histopathological and Morphometric Analyses

**Figure 11:**
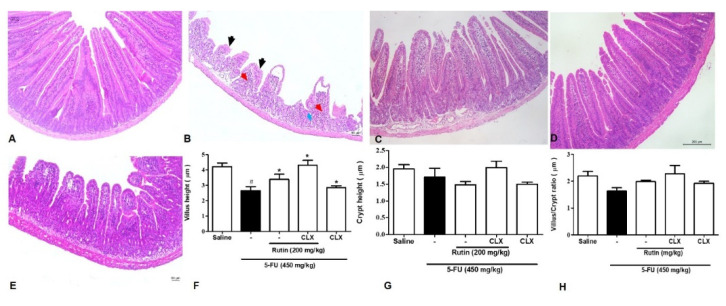
Effect of RUT on the COX-2 pathway evaluated by histopathological and morphometric analysis in jejunal sections. hematoxylin and eosin (H&E) staining was performed for morphometric and histopathological analysis of tissues after incubation with celecoxib. Saline (**A**); 5-FU (**B**); RUT-200 (**C**); CLX (**D**); RUT and CLX (**E**); Height of villi (**F**); Depth of crypts (**G**); Villus/Crypt ratio (**H**). The histopathological issues are indicated by arrows. Inflammatory cell infiltration (red arrow), decreased intestinal villi (black arrow), and loss of intestinal crypt architecture (blue arrow). Values are expressed as mean ± SEM. For statistical analysis, one-way ANOVA was used, followed by the Tukey test. # *p* < 0.05 vs. saline group, * *p* < 0.05 vs. 5-FU group. 5-FU, 5-fluorouracil; RUT, rutin.

As illustrated in Figure 11F, 5-FU significantly reduced the jejunal villi height when compared with the saline group (*p* < 0.05). Conversely, 200 mg/kg RUT reversed the reduced villi height when compared with 5-FU treatment. Treatment with the COX-2 inhibitor, celecoxib (CLX), significantly suppressed a decrease in villi height when compared with the 5-FU group. Compared with the 5-FU treatment, 200 mg/kg RUT in combination with CLX could prevent a decrease in villi height (*p* < 0.05). The group treated with a combination of 200 mg/kg RUT and CLX exhibited a greater extent of reversal of decreased villi height when compared with groups treated with monotherapy of RUT 200 or CLX (*p* < 0.05).

Regarding the jejunal crypts and the villus/crypt ratio in Figure 11G,H, no significant differences were observed between the 5-FU and the saline group (*p* < 0.05). Additionally, no significant difference was observed between the 5-FU group and the 200 mg/kg RUT and/or CLX groups.

The photomicrographs are presented in Figure 11A–E, demonstrating that 5-FU reduced villi height in addition to impairing villi and crypt architecture, inducing edema, and increasing inflammatory infiltrates when compared to the saline group. Furthermore, RUT 200 mg/kg and/or CLX treatment groups exhibited reduced levels of 5-FU-induced histopathological damage.

### 2.8. Immunohistochemistry for the Detection of COX-2 Activity

**Figure 12:**
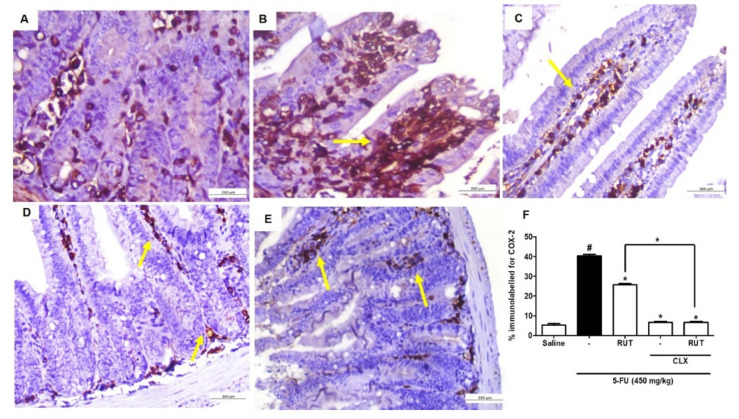
Immunohistochemical analysis of COX-2 expression. Saline (**A**); 5-FU (**B**); RUT-200 (**C**); CLX (**D**); RUT and CLX (**E**). % immunolabelled for COX-2 (**F**). Values are expressed as mean ± SEM. For statistical analysis, one-way ANOVA was used, followed by Tukey’s test. # *p* < 0.05 vs. saline group, * *p* < 0.05 vs. group 5-FU.

Using immunohistochemical analyses, we investigated the effects of 200 mg/kg RUT in the presence or absence of CLX on the COX-2 expression levels in 5-FU-induced intestinal mucositis. 5-FU promoted the intense immunostaining of COX-2 (Figure 12B) in the jejunal mucosa when compared with the saline group (*p* < 0.05) (Figure 12A,F). As shown in Figure 12C, pretreatment with 200 mg/kg RUT decreased COX-2 immunostaining when compared with the 5-FU group (*p* < 0.05). Similarly, CLX alone (Figure 12D) or the CLX and RUT 200 mg/kg combination (Figure 12D,E) decreased COX-2 immunostaining in mice presenting 5-FU-induced intestinal mucositis, when compared with the 5-FU group (*p* < 0.05).

### 2.9. Molecular Docking

**Figure 13:**
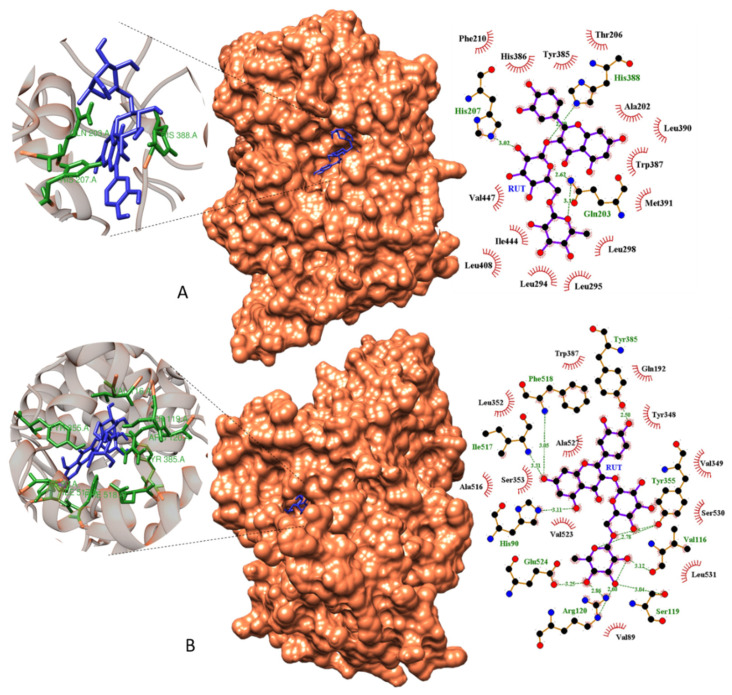
Molecular docking models of RUT with COX-1 (**A**) and COX-2 (**B**). Surface docking poses and Ligplot 2D diagrams show details of hydrogen bonds and hydrophobic interactions in complexes RUT/COX-1 (**A**) and RUT/COX-2 (**B**). RUT, rutin; COX, cyclooxygenase.

To investigate the underlying protective mechanisms of RUT against the 5-FU-induced intestinal mucositis, we performed a molecular docking analysis of the potential RUT target sites with COX-1 and COX-2 enzymes. Molecular docking of the RUT complex/COX-1 revealed a binding energy of −7.28 Kcal mol^−1^ and inhibition constant of 4.62 µM. Three hydrogen bond interactions at Gln203, His207, and His388 amino acid sites; the Gln203 residue demonstrated two hydrogen bond interactions at distances of 2.62 and 3.10 Å (Figure 11A). This site revealed the most concentrated molecular interactions of the complex, with high interaction energy near the edge of the active site of the receptor. The amino acids Thr206, Phe210, His386, Tyr385, Ala202, Leu390, Trp387, Met391, Leu298, Leu294, Leu295, Leu408, Ile444, and Val447 participated in the hydrophobic interactions (Figure 13A).

In the biological potency studies, the most attractive target observed was Cox-2, where RUT exhibited a high molecular affinity when coupled to the active site of the receptor, with a −10.07 Kcal.mol^−1^ binding energy and an inhibition constant of 41.682 nM. This molecular affinity was expressed with the nine hydrogen bonds formed in amino acids Arg120, Glu524, His90, Ile517, Phe518, Ser119, Tyr355, Tyr385, and Val116, acting directly at the receptor binding site, which was the most vulnerable Cox-2 site (Figure 13B). The amino acids Trp387, Leu352, Ala516, Ser353, Val523, Ala527, Val89, Leu531, Ser530, Val349, Tyr348, and Gln192 were responsible for the hydrophobic interactions between the receptor and the ligand (Figure 13B).

## 3. Discussion

The assignments of Infrared Absorption Spectroscopy (FTIR) and ^1^H and ^13^C-NMR suggested that RUT was the main flavonoid extracted from the beans of *D. gardneriana*. On the other hand, the two endothermic peaks shown in the DSC curve can be justified by the occurrence of polymorphisms in the crystalline structure of the RUT, as reported in the literature [34]. However, the isolation of RUT as a major compound was only confirmed with HPLC, based on retention time and UV-Vis spectrum data. The results obtained are in line with those reported by other authors [35,36].

Flavonoids represent a highly diverse class of secondary metabolites, with various biological activities demonstrating beneficial properties on the gastrointestinal system, such as antispasmodic, antisecretory, antidiarrheal, and antiulcer properties [17,18,37]. In the present study, we reported the effects of RUT on 5-FU-induced intestinal mucositis, demonstrating that 200 mg/kg RUT could reverse the deleterious effects of an antineoplastic agent, 5-FU, on the intestines, including oxidative damage, neutrophil recruitment, mastocytosis, depletion of goblet cells, and histological and morphometric alterations.

During 5-FU chemotherapy, weight loss is considered a common side effect. Therefore, body mass assessment is one of the parameters evaluated daily to assess whether intestinal mucositis was induced by 5-FU. Similar to our results, previous studies have reported a decrease in animal body weight following 5-FU-induced intestinal mucositis [38,39,40]. In the present study, we demonstrated that RUT failed to reverse the weight loss induced by 5-FU. Furthermore, weight loss is a side effect that activates inflammatory responses followed by gastrointestinal dysfunction [41]. Therefore, RUT could prevent morphophysiological changes in mucositis by mechanisms independent of weight loss.

Histopathological alterations, such as decreased villi height, crypt necrosis, edema, vacuolization, inflammatory infiltrates, and loss of architecture, are typically reported as adverse effects of 5-FU. In the present study, we demonstrated the histological changes stated above following the induction of intestinal mucositis in mice. Furthermore, RUT prevented histopathological alterations at the 200 mg/kg dose. The protective abilities of RUT in the gastrointestinal tract have been reported in the literature [42,43] at doses similar to those used in the present study, corroborating the results obtained.

In addition to the histopathological alterations, myelosuppression in the form of leukopenia is a typical adverse effect reported extensively following the use of antineoplastic agents. In the present study, 5-FU induced intense leukopenia, as reported by Soares et al. [44] and Quaresma [45], following single-dose 5-FU (450 mg/kg) administration; however, RUT could reverse the adverse effects at all test doses. Flavonoids act via different mechanisms to attenuate the damaging effects of mucositis.

Regarding the MPO concentrations, we observed that RUT prevented increased MPO levels in the jejunum induced by 5-FU treatment. Similarly, Bastos et al. [46], Justino et al. [47], and De Ávila et al. [48] have reported increased MPO activity following the induction of intestinal mucositis using 5-FU. MPO has been used as a quantitative marker of neutrophil infiltration in various organs, including the gastrointestinal tract. Moreover, the inflammatory properties in other lesion models in the gastrointestinal tract, as well as different systems, have reported the anti-inflammatory potential of RUT [42,49,50,51].

RUT, like many flavonoids, has demonstrated antioxidant properties [52], in addition to the ability to relieve oxidative stress in biological systems. We observed that RUT exhibited antioxidant effects against intestinal mucositis, increasing GSH levels and decreasing MDA levels in mice presenting 5-FU-induced intestinal mucositis; this was consistent with the findings of previous studies that demonstrated that RUT exerts antioxidant effects by decreasing MDA levels [53,54] and increasing GSH and superoxide dismutase levels [55,56,57].

In addition to causing an antioxidant imbalance and exacerbating inflammation, mucositis promotes the disorganization of tissue architecture and, in turn, alters the proportions of intestinal mucosa resident cells, such as mast cells and goblet cells, which are critical for the maintenance of intestinal epithelial homeostasis.

In the present study, RUT reversed the increase in mast cell numbers induced by 5-FU. Flavonoids act via several mechanisms of action, and as reported in previous studies, this class of biomolecules can interfere with the release of histamine and proton pump activity, as is the case with quercetin [17].

Mast cells are powerful immunological modulators of the tissue microenvironment, which act in a distinct manner, depending on the nature of surface receptors and mediators involved. They facilitate gastrointestinal homeostasis, through immune protection, regulation of the architecture and permeability of the epithelial barrier, and remodeling of mucous tissue, by stimulating fibroblast growth [58]. Conversely, they are considered critical in the pathogenesis of inflammatory processes based on their overexpression, which amplifies the inflammatory response due to the selective release of mediators [59,60]. When activated, the cells release a variety of biologically active products, followed by a wave of synthesis and secretion from the mediator [61]. Consequently, by attenuating the increase in mast cell count, the protective effect of RUT was evident in intestinal mucosa and submucosa lesions induced by 5-FU. The data are consistent with results presented by De Miranda et al. [62], where a decrease in mast cells was observed following the administration of troxerutin, a flavonoid derived from RUT, in mice with 5-FU-induced intestinal mucositis.

In the present study, a decrease was observed in the number of goblet cells induced by 5-FU when compared to the saline group. Additionally, RUT attenuated goblet cell loss in the intestinal mucosa. Carneiro-Filho et al. [63], Stringer et al. [64], and Gawish et al. [65] have reported that chemotherapy with 5-FU mainly causes a marked disturbance in the membranes at brush borders and absorptive dysfunction, largely due to a decrease in the number of goblet cells in the intestinal mucosa. In addition to the absorptive dysfunction induced by 5-FU, a decrease in the number of goblet cells and deregulation mucus release occurs, which, in turn, would result in the depletion of mucus storage by residual goblet cells, as well as premature stem cell death, which reflects the renewal of all cell lines, including goblet cell lines, as reported by Gawish et al. [65]. Consequently, RUT potentially promotes mucosal protection by attenuating goblet cell loss and enhancing the integrity of the mucous barrier and its absorptive capacity.

In gastrointestinal disorders, the therapeutic effects of flavonoids are largely attributed to their antioxidant and anti-inflammatory properties. In terms of anti-inflammatory activity, flavonoids can inhibit cyclic adenosine monophosphate and COX activity, as well as protein phosphorylation [66,67,68,69,70]. In the case of RUT, its anti-inflammatory effects have been attributed to its capacity to suppress the production of tumor necrosis factor-alpha (TNF-α) and interleukin 6 (IL-6), as well as its ability to activate nuclear factor B (NF-kB) and extracellular kinases [71]. Therefore, in the molecular docking and histopathological analyses performed to investigate the interaction between RUT and CLX, and COX-1 and 2 enzymes, RUT stably bound to the target sites of COX-2 and COX-1 (in decreasing order of interaction, respectively).

In the histological analysis, in addition to observing that RUT (200 mg/kg) reversed the deleterious effects of 5-FU, CLX reversed villi shortening and increased the villus/crypt ratio. Furthermore, pretreatment with RUT in combination with CLX led to a superior reversal of the morphometric alterations than with pretreatment with RUT (200 mg/kg) or CLX individually. In COX-2 immunohistochemistry analyses, a decrease in the proportion of immunostaining was observed following treatment with RUT, CLX, and RUT in combination with CLX.

**Figure 14:**
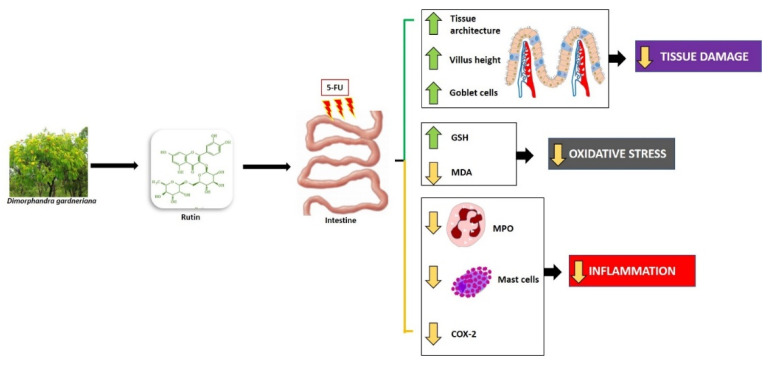
Hypothetical chemopreventive effect of RUT in intestinal mucositis induced by 5-FU. RUT prevents intestinal inflammation by inhibiting MDA, MPO, COX-2, oxidative stress, mastocytosis. RUT also stimulates increased villi and increased levels of the antioxidant GSH. RUT, Rutin; COX-2, Cyclooxygenase 2; MDA, Malondialdehyde; MPO, Myeloperoxidase; GSH, Reduced Glutathione. Green arrows (stimulate/increase), yellow arrows (inhibit).

Considering that COX-2 plays a key role in the management of inflammation through the release of arachidonic acid, and, in turn, eicosanoid biosynthesis, as an example of a prostaglandin involved in various immune and inflammatory responses, the results obtained in the present study are consistent with our understanding of CLX and COX-2 functions against intestinal inflammatory disorders. According to Short et al. [72], low CLX doses could be therapeutically utilized to protect the intestinal barrier in patients with inflammatory bowel disorders, owing to its capacity to reduce COX-2 expression. Javle et al. [73] have reported that a combination of irinotecan and CLX has demonstrated antitumor effects, with an improvement in irinotecan-induced diarrhea and mortality. Additionally, RUT (200 mg/kg) monotherapy or in combination with CLX could decrease 5-FU-induced COX-2 expression in the jejunum. The results observed in the present study are consistent with the findings of Gawish et al. [65], who observed that RUT could inhibit the enzymatic activity of COX-1, COX-2, and 15-lipoxygenase (15-LOX) with 75.63, 81.00, and 80.43% inhibition, respectively. Therefore, they proposed that the pharmacological action of RUT in 5-FU-induced intestinal mucositis occurs as illustrated in Figure 14.

As observed in our work, other studies investigating the effect of flavonoids such as luteolin, quercetin and troxerutin on intestinal mucositis promoted by antineoplastic agents, indicated, among the possible causes of protective and therapeutic effect, the anti-inflammatory, antioxidant and inhibition properties of apoptosis, and consequently the ability to promote tissue recovery [62,74,75].

## 4. Materials and Methods

### 4.1. Extraction and Characterization of the RUT Flavonoid

*D. gardneriana* beans were collected in the city of Crato in the state of Ceará, Brazil. The aerial parts of plants were deposited at the Prisco Bezerra Herbarium under the accession number 32339. RUT was extracted from the *D. gardneriana* beans and characterized according to Vila-Nova et al. [76]. *D. gardneriana* beans (150 g) were added to a Soxhlet extractor and RUT was extracted with hexane, ethyl acetate, methanol, and water. The solvents were concentrated in a rotary evaporator. Hexane (0.38 g), ethyl acetate (4.47 g), methanol (47.53 g), and aqueous (8.49 g) extracts were obtained. After extract analysis using TLC, the ethyl acetate and methanol extracts were combined and dispersed in 200 mL of cold water. After stirring, the mixture was filtered and the resulting residue was washed with an additional 100 mL of water. After filtering again, the residue was dried in a 100 °C oven, yielding 18.0 g of a yellow powder. The material was subjected to column chromatography on a silica gel column, and then eluted with mixtures of hexane, dichloromethane, ethyl acetate, and methanol with increasing polarity. The fractions were collected and compared by TLC. This method resulted in the purification of 12.42 g of RUT. Spectral data were compared to those found in the literature [77].

#### 4.1.1. Infrared Absorption Spectroscopy (FTIR)

The FTIR spectrum was obtained with a Vertex 70v spectrometer, Bruker (San Diego, CA, USA), using KBr tablets, in a spectral region between 4000 and 500 cm^−1^, with a resolution of 2 cm^−1^ from 128 scans.

#### 4.1.2. Differential Scanning Calorimetry (DSC)

Approximately 5.0 mg of RUT was weighed and added to a hermetically sealed aluminum pan. The analysis was carried out under the following conditions: nitrogen atmosphere with flow rate of 50 mL min^−1^, heating ramp from 25 to 500 °C and heating rate of 10 °C min^−1^ in a DSC 50 Shimadzu equipment (Kyoto, Japan).

#### 4.1.3. Nuclear Magnetic Resonance (NMR)

The ^13^C-NMR and ^1^H-NMR data were obtained using the Fourier transform Bruker Avance-DRX 500 spectrometer (San Diego, CA, USA), equipped with an inverse detection probe operating at a frequency of 125 MHz (^13^C) and 499.9 MHz (^1^H). The experiment was performed by dissolving 20.0 mg of RUT in 0.6 mL of deuterated dimethyl sulfoxide (DMSO-D6). The analysis was carried out in 5 mm tubes and the chemical shifts (δ) were expressed in ppm.

#### 4.1.4. High Performance Liquid Chromatography (HPLC)

Chromatographic analysis of RUT was performed using a Shimadzu LC-10AD pump (Kyoto, Japan), Shimadzu SPD-M10AVP (Kyoto, Japan) photodiode array detector. The methodology proposed by [35] with some modifications was used. The mobile phase was prepared using an isocratic system, consisted of a solution of acetonitrile/phosphoric acid pH 3 (20:80, *v*/*v*), which was injected in a flow of 1.2 mL min^−1^ at a temperature of 40 °C. For the determination of RUT, a wavelength of 350 nm and a HIBAR chromatographic column (250 × 4.6 mm, 5 µm) were used. It was possible to determine the purity of RUT by normalizing the area, as well as identifying it through the scanning spectrum in the UV-Vis region and retention time.

### 4.2. Drugs and Reagents

Two drugs were used for mucositis induction and treatment, respectively: 5-FU (FauldFluor^®^, Libbs, São Paulo, Brazil) celecoxib (CLX-Celebra^®^, Pfizer, São Paulo, Brazil). RUT was dissolved in distilled water with Tween 20 (1%) before use. All drugs and reagents were prepared immediately before use.

### 4.3. Animals

The animals were obtained from the Department of Surgery at the Federal University of Ceará (UFC). Male Swiss mice (25–30 g) were housed in polypropylene cages, lined with wood, in a controlled environment with a temperature of 23 ± 2 °C, at a 12 h light/12 h dark cycle, with free access to water and standard feed. The procedures and experimental protocols were approved by the Ethics Committee on Animal Use of the Federal University of Ceará (CEUA-UFC) under number 6595260719.

### 4.4. Experimental Protocol of 5-FU-induced Intestinal Mucositis

The experimental model of intestinal mucositis was established using Swiss mice as described by De Miranda et al. [62]. 5-FU (450 mg/kg) was administered intraperitoneally (i.p) as a single dose on the first day of the experimental protocol. To evaluate the effective dose of RUT against 5-FU-induced morphological changes, 50, 100, and 200 mg/kg RUT were administered orally on the first, second, and third days, respectively. The first dose of RUT was administered 1 h before 5-FU injection, whereas, the second and third doses were administered 24 and 48 h after 5-FU injection, respectively. On the fourth day of the experimental protocol, the animals were euthanized using an anesthetic overdose of ketamine and xylazine (270 and 15 mg/kg, respectively). Intestinal samples were collected. To confirm the experimental model of 5-FU induced intestinal mucositis, mice body weights were assessed daily before the administration of treatment. In the current study, RUT doses were considered in accordance with those in previously published studies [42,65,78,79,80].

With the analysis of the parameters (weight analysis, histopathological and morphometric), the effective dose of RUT in the treatment of intestinal mucositis and the intestinal segment with the best response to treatment with RUT were determined. Starting from the best dose of RUT (200 mg/kg), the evaluation of the other parameters (mast cell and goblet cell count, MPO, GSH and MDA measurements) was continued, as well as the modulation of the COX-2 pathway.

To investigate the role of COX-2 during RUT treatment in 5-FU-induced intestinal mucositis, COX-2 was blocked by an i.p. injection of 7.5 mg/kg CLX. Starting with the effective dose of RUT (200 mg/kg) for the treatment of intestinal mucositis, a 5-FU mucositis induction protocol was initiated, similar to the first investigation. Here, mice were divided into three treatment groups, i.e., RUT-200 (200 mg/kg orally), CLX (7.5 mg/kg, i.p), and RUT + CLX (RUT: 200 mg/kg orally and CLX: 7.5 mg/kg, i.p), and two control groups, saline and 5-FU. Overall, during the current study, mice were randomly divided to the following groups (*n* = 6 in each group): Saline (0.9% NaCl), 5-FU (450 mg/kg of 5-FU + 0.9% NaCl), RUT-50 (5-FU + 50 mg/kg RUT), RUT-100 (5-FU + 100 mg/kg RUT), RUT-200 (5-FU + 200 mg/kg RUT), CLX (5-FU + 7.5 mg/kg of CLX), and RUT + CLX (5-FU + 200 mg/kg RUT + 7.5 mg/kg CLX).

### 4.5. Histopathological and Morphometric Analysis

After euthanasia, intestinal samples were collected and fixed in 10% formaldehyde for histopathological and morphometric analysis [44,81]. These samples were embedded in paraffin, sectioned to 4 μm slices, and stained with H&E. A blinded and randomized histopathological analysis was performed by an experienced histopathologist to assess the severity of mucositis using a scoring system [82]. Tissues ranged from 0 (no lesion/normal histological findings) to 3 (maximal grade lesion), indicating shortened villi, vacuolated cells, crypt necrosis, intense inflammatory cell infiltration, vacuolization and edema in the mucous and muscle layers with edema, vacuolization, and neutrophilic infiltrate. The effective dose of RUT for the treatment of intestinal mucositis was determined based on the histological analysis, leukocyte count, and weight measurement.

### 4.6. MPO Assay

MPO activity was determined by the technique described by Bradley et al. [83]. Briefly, samples from the jejunal segment (50–100 mg), corresponding to the animals in the saline, RUT 200 mg/kg, and 5-FU (50–100 mg) groups, were homogenized in 1 mL potassium buffer containing 0.5% hexadecyltrimethylammonium bromide (HTAB) and centrifuged (4000 rpm, 7 min, 4 °C). MPO activity was analyzed by measuring absorbance at 450 nm using diisocyanate dihydrochloride and 1% hydrogen peroxide in the resuspended pellet. The results were recorded as MPO units per mg of tissue.

### 4.7. Measurement of GSH and MDA Levels

For estimating GSH and MDA levels, samples from the jejunal segment were obtained from the animals in the saline, RUT-200, and 5-FU groups, and were homogenized in cold EDTA or KCl (1:9, *v/p*) to prepare a 10% homogenate suspension. The GSH levels were estimated according to the method described by Sedlak and Lindsay [84], with minor modifications. Aliquots (400 μL) of homogenized tissue were mixed with 320 μL distilled water and 80 μL trichloroacetic acid (50%, *w/v*) and centrifuged at 3000 rpm for 15 min. The supernatant (400 μL) was mixed with 800 μL Tris buffer (0.4 M, pH 8.9), followed by the addition of 5,5-dithiobis (2-nitrobenzoic acid) (DTNB; 0.01 M). GSH absorbance was read at 405 nm and its concentration was expressed in μg/mg tissue. Conversely, lipid peroxidation was determined by assessing the level of thiobarbituric acid reactive substances (TBARS) measured as MDA [85]. The homogenates were incubated at 37 °C for 1 h and added to 400 μL of 35% perchloric acid. The mixture was centrifuged (5000 rpm, 10 min at 4 °C) and 400 µL of 0.6% thiobarbituric acid was added to the supernatant, followed by incubation at 98 °C for 1 h. After cooling, the MDA absorbance was read at 532 nm and its concentration was expressed as nmol/mg tissue.

### 4.8. Intestinal Mucosa Cell Count: Goblet and Mast Cells

To enable the identification and quantification of mast cells and mucus-secreting cells (goblet), the paraffin blocks with jejunal segment samples, corresponding to the saline, RUT-200, and 5-FU groups, were selected for toluidine blue staining, according to Michalany et al. [86] and periodic acid Schiff (PAS) according to Sano et al. [87]. Staining was performed after de-paraffinization of the slides with xylol, followed by hydration with absolute alcohol, and a series of 90%, 80%, and 70% alcohol dilutions. Then, the slides were washed with distilled water, stained with toluidine blue for 8 min, washed, and dried. For PAS, the slides were incubated in periodic acid, the Schiff reagent, and Carazzi hematoxylin dyes for 1, 10, and 10 min, respectively, followed by successive washing with distilled water, as recommended by EasyPath^®^ (Erviegas, Indaiatuba, SP, Brazil). To enumerate mast and goblet cells present on slides, with the aid of an optical microscope and image acquisition system (LEICA, Wetzlar, Germany), digital images were captured for subsequent counting of at least 10 fields, using the ImageJ^®^ software version 1.8.0 (National Institutes of Health, Bethesda, MD, USA). Results represent the average of 10 fields from each group.

### 4.9. Immunohistochemistry for the Detection of COX-2

The jejunal sections were deparaffinized in an oven at 60 °C, followed by three cycles of xylol immersion for 5 min each. Then, the sections were rehydrated in decreasing alcohol concentrations (100, 90, 80, and 70%). Next, the histological sections were washed with distilled water for 10 min, followed by antigenic recovery in citrate buffer (pH 7.0, DAKO^®^, São Paulo, Brazil) for 20 min in a water bath (95 °C). The slides were then washed with phosphate-buffered saline solution (PBS) for 5 min at room temperature. Next, endogenous peroxidase blockade was performed with 3% hydrogen peroxide solution (H_2_O_2_) for 30 min. The sections were then incubated overnight with goat anti-COX-2 primary antibody (SantaCruz^®^, Dallas, TX, USA), diluted in antibody diluent (1:100) for 60 min. After the slides were washed with PBS and incubated with rabbit IgG (GBI Labs^®^, Bothell, WA, USA), the secondary antibody was diluted (1:400) for 30 min. For visualization, the sections were incubated with the streptavidin-conjugated peroxidase complex (ABC complex) for 30 min and chromogen 3,30 diaminobenzidine peroxide, DAB (DAKO^®^, São Paulo, Brazil), followed by counterstaining with hematoxylin (DAKO^®^, São Paulo, Brazil) for 10 min. Simultaneously, negative controls were processed as described above, with the primary antibody replaced with the antibody diluent. The procedures were performed in an automated manner using Autostainer Plus (DAKO^®^, São Paulo, Brazil). For the COX-2 immunostaining images, quantification was performed by measuring the % immunolabelled area with the aid of Adobe Photoshop10. All images were captured using an optical microscope and an image acquisition system (LEICA, Wetzlar, HE, Germany).

### 4.10. Molecular Docking and Determination of RUT Binding Sites

The 3D structures of COX-1 and COX-2 enzyme targets were obtained from the PDB protein database (Protein Data Bank, 2019). Molecular docking calculations were performed using the Autodock 4.2^®^ program [88,89,90]. Proteins and ligands were prepared for molecular docking using the Autodock Tools (ADT) version 1.5.6 program. The receptor was considered rigid, while each ligand was considered flexible. The Lamarckian Genetic Algorithm (LGA) with global search and pseudo-Solis and Wets with local search methods were used for molecular docking, and 100 independent runs were performed for each simulation [91]. The remaining docking parameters were set to default values. Molecular docking analyses focused on the low-energy clusters, and the conformation with the lowest energy combined with visual inspection was chosen for detailed analysis.

### 4.11. Statistical Analysis

For parametric distribution, data are expressed as mean ± SEM; for non-parametric distribution (e.g., histological scores), data are expressed as the median. Data normality was analyzed using the Shapiro–Wilk test.

The results demonstrating a parametric distribution were analyzed using ANOVA, followed by the post hoc Tukey test, using GraphPad Prism version 6.0 (GraphPad Software Inc., La Jolla, CA, USA). The data presenting non-parametric distribution were analyzed using the Kruskal–Wallis test, followed by Dunn’s test (multiple comparisons). A *p*-value of <0.05 was considered statistically significant.

## 5. Conclusions

The chemical characterization showed that RUT was the main flavonoid extracted from the *D. gardneriana’s* beans with 97% purity. In summary, RUT prevented functional and inflammatory changes induced by 5-FU in intestinal mucositis, observed as the reversal of histopathological and morphometric changes, oxidative damage, neutrophilic infiltration, mastocytosis, and goblet cell depletion. The effects of RUT are likely to be mainly associated with the COX-2 pathway, directly and indirectly (through the inhibition of transcription factors such as NF-kB, interleukins, and pro-inflammatory enzymes), based on a decrease in COX-2 immunostaining and molecular docking that revealed the binding affinity between RUT and COX-2 binding sites. However, further studies are required to elucidate the underlying molecular mechanisms of RUT effects following the expression of pro-inflammatory cytokines, in addition to evaluating other potential mechanisms by which RUT prevents chemotherapy-induced intestinal mucositis.

## Figures and Tables

**Table 1:** ^1^H Nuclear Magnetic Resonance (NMR) and ^13^C-NMR profile of RUT (DMSO-*d*_6_, δ, multiplicity).

^1^H-NMR		^13^C-NMR	
Rutin	δ (ppm)	Rutin	δ (ppm)
5-OH	12.6 (s)	4-C	177.8
2′-Ar	7.5 (s)	7-C	164.5
5′-Ar	6.8 (m)	9-C	161.7
8-Ar	6.4 (s)	5-C	157.1
6-Ar	6.2 (s)	2-C	156.9
1″-H	5.4 (d)	3′-C	148.9
1‴-H	4.4 (s)	4′-C	145.2
9H-rhamnoglucosyl	3.7–3.1	3-C	133.8
3H-rhamnosyl	1.0 (m)	1′-C	122.0
		6′-C	121.6
		5′-C	116.7
		2′-C	115.7
		1″-C	101.6
		1‴-C	101.2
		6-C	99.1
		8-C	94.0
		3″-C	76.9
		5″-C	76.4
		2″-C	74.5
		4‴-C	72.3
		2‴-C	71.0
		4″-C	70.8
		3‴-C	70.5
		6″-C	68.7
		5‴-C	67.4
		6‴-C	18.2

**Table 2:** Histopathological scores of mice subjected to 5-FU-induced intestinal mucositis and pretreated with RUT.

Segments	Groups				
	Saline	5-FU	RUT (mg/kg)		
			50	100	200
Duodenum	0 (0–1)	3 (2–3) ^a^	2 (1–3)	2 (1–2)	1 (1–3) ^b^
Jejunum	0 (0–1)	3 (1–3) ^a^	2 (1–3)	1.5 (1–3)	1 (1–2) ^b^
Ileum	0 (0–0)	3 (1–3) ^a^	3 (1–3)	2 (1–3)	2 (1–3)

Values are expressed as median, where ^a^ *p* < 0.05 vs. saline and ^b^ *p* < 0.05 vs. 5-FU (*n* = 6/group). The data were analyzed using the Kruskal–Wallis test followed by Dunn’s multiple comparisons test. 5-FU, 5-fluorouracil; RUT, rutin.
